# Chemotherapy plus targeted drugs in conversion therapy for potentially resectable colorectal liver metastases: a meta-analysis

**DOI:** 10.18632/oncotarget.9675

**Published:** 2016-05-27

**Authors:** Lu Wang, Yinan Sun, Ben Zhao, Huixian Zhang, Qianqian Yu, Xianglin Yuan

**Affiliations:** ^1^ Department of Oncology, Tongji Hospital, Huazhong University of Science and Technology, Wuhan, Hubei Province, China

**Keywords:** colorectal liver metastases, conversion therapy, targeted drug, surgery, meta-analysis

## Abstract

**Objectives:**

To evaluate the safety and efficiency of the conversion therapy : chemotherapy plus anti-epidermal growth factor Receptor (EGFR) or anti-vascular endothelial growth factor receptor (VEGFR) monoclonal antibodies (MoAbs) with different rat sarcoma (RAS) status in patients with potentially resectable colorectal liver metastases (CRLM).

**Methods:**

Randomized controlled trials (RCTs) were identified and the association between RAS mutation and clinical outcome in CRLM patients treated with anti-EGFR or anti-VEGFR MoAbs was investigated. Searches were performed for data recorded between January 2005 and August 2015 in the Cochrane Library, MEDLINE, PubMed, and EMBASE. Objective response rates (ORR), conversion resection rates (CRR), R_0_ resection rates (R_0_R) and rate ratios (RR) were used to assess the strength of the association between different RAS status, MoAbs and conversion efficiency.

**Results:**

In the conversion therapy, ORR and RR were associated with patients with wild type RAS and different MoAbs. Patients treated with MoAbs: anti-VEGFR or anti-EGFR drugs, resulted in higher ORR, (RR=1.53, 95% confidence interval [CI]: 1.27-1.84, *P* < 0.05). Furthermore, anti-EGFR regimens displayed higher ORR compared with anti-VEGFR regimens in CRLM patients, (RR=1.15, 95%CI: 1.04-1.26, *P* < 0.05). However, CRLM patients with mutant type RAS did not benefit from anti-EGFR therapy, (RR=0.91, 95%CI: 0.76-1.08, *P*<0.05) and wild type RAS patients displayed higher ORR with anti-EGFR therapy, (RR=1.56, 95%CI: 1.16-2.01, *P* <0.05). In addition, the patients achieved higher resection rates (RR=1.67, 95%CI: 1.00-2.81, *P* ≤ 0.05) and R_0_ resection (RR=1.85, 95%CI: 1.04-3.27, *P* < 0.05).

**Conclusion:**

We noted that the addition of MoAbs (anti-EGFR or anti-VEGFR) to standard chemotherapy could improve conversion efficiency for patients with potentially resectable CRLM patients, and anti-EGFR therapies maybe more effective than anti-VEGFR therapies. RAS status is a potential predictive marker of the clinical benefit resulting from treatment with anti-EGFR MoAbs therapy in CRLM patients and anti-EGFR MoAbs therapy could displayed greater efficiency only in patients with wild type RAS.

## INTRODUCTION

Colorectal cancer is the third most commonly diagnosed cancer in males and the second in females, with an estimated 1.4 million cases and 693,900 deaths globally per year [1]. At least 50% of patients develop metastases [2]. Fewer than 12.9% of patients with stage 4 colorectal cancer had a 5-year survival [3]. Only a few patients with liver-only metastatic disease had a 30-60% chance of survival 5 years after surgical resection [4]. This stark difference in 5-year survival highlighted the potential benefits of liver resection. However, the majority patients have unresectable tumors [5], and preoperative therapy may raise the hope of these patients. Preoperative therapy is administered in either of two settings: neoadjuvant therapy is administered preoperatively for initially CRLM, making the resection from R_x_ to R_0_ (R_x_ resection: presence of residual tumor cannot be assessed, R_0_ resection: macroscopically complete removal by non-contaminated surgery with wide or radical margin, R_1_ resection: microscopic residual tumor) [10], and conversion therapy is given to patients with initially unresectable disease in an attempt to render the patient's disease resectable [12]. Systemic conversion therapy may include combinations of chemotherapeutic agents (irinotecan or oxaliplatin-based), with or without the use of targeted MoAbs targeting (anti-VEGFR: bevacizumab or anti-EGFR: cetuximab and panitumumab). The efficiency of the targeted therapy is associated with the gene status and the identification of the correlation between a mutated cancer gene and the response to a targeted therapy is complex. This may be due to intratumor heterogeneity [13]. Different RAS type tumors should be evaluated especially in patients administered anti-EGFR MoAbs. The efficiency of conversion therapy depends upon response rate and R0 resection, and a strong relationship between the tumor rate ratio (RR) and resection rates has been demonstrated in patients with CRLM treated with chemotherapy [12]. Although the conversion therapy could provide benefits for the patients with unresectable CRLM, the preferred choice of MoAbs for conversion therapy remains debatable, and there has been no randomized study specifically investigating patients with borderline resectable CRLM [7]. Therefore, the choice of standard chemotherapy to be administered, and whether or which type of MoAbs should be used in conversion therapy has yet to be elucidated by RCTs and need to be explored.

## MATERIALS AND METHODS

### Literature review

The intervention measures in the present meta-analysis involved a comparison of the three groups follows: anti-VEGFR MoAbs plus chemotherapy *vs*. anti-EGFR MoAbs plus chemotherapy *vs*. placebo (or blank control) plus chemotherapy prior to the surgical resection. Searches were performed for data recorded between January 2005 and August 2015 in the Cochrane Library, MEDLINE, PubMed, and EMBASE. The search terms were: “Conversion therapy”, “CRLM” and “MoAbs”. The inclusion criteria were histologically established unresectable CRLM by pathology or imaging; 0-2 points according to Eastern Cooperative Oncology Group (ECOG) score standard; no detectable extrahepatic tumors; and age of 18-80 years. The exclusion criteria were: non-randomized controlled trials; use of neoadjuvant therapy; and failure to comply with any of the above inclusion criteria.

### Quality assessment

Two reviewers (WL and SYN) reviewed independently to assess the eligibility of each study for inclusion. Any discrepancy in the selection of studies or data retrieved by the reviewers was resolved by discussion with a third reviewer. The quality of the included literatures was appraised and graded according to PRISMA Checklist and a modified Jadad scale [14] as follows: low quality, 1-3 points; and high quality 4-7 points. Others were excluded to reduce the influence of the critical analysis result for the RCTs with high statistical power and consistent reporting, with a score of 3 considered acceptable and a higher score indicating better reporting.

### Data extraction and analysis

Objective response rates (ORR), conversion resection rates (CRR), R_0_ resection rates (R_0_R) and RR were selected for the statistical analysis of the data. The analyses were defined prospectively in a statistical analysis plan. The heterogeneity analysis was performed prior to combining data (heterogeneity was quantitatively analyzed using I^2^): heterogeneity was considered mild when *I*^2^ < 25%; moderate when 25% ≤ *I*^2^ < 50%; great when 50% ≤ *I*^2^ < 75%; and high when 75% ≤ *I*^2^ < 100%. The fixed analysis was performed using RevMan 5.2 software on the data with low heterogeneity; Random analysis was performed for the data with higher heterogeneity. All reported 95% confidence intervals (CIs) and *p* values following a two-sided test with a value of *p* ≤ 0.05 were considered indicative of statistical significance.

## RESULTS

### Included studies

**Figure 1:**
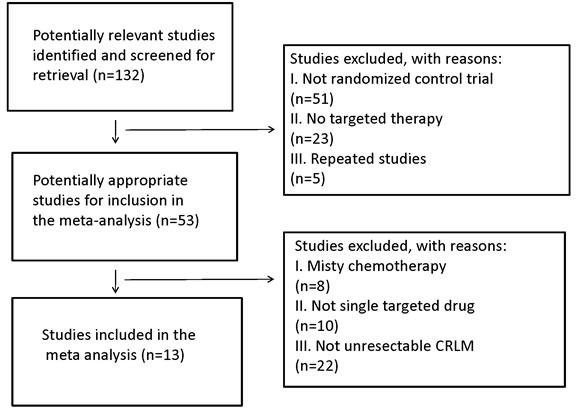
Search strategy and flow chart for the present meta-analysis

A total of 132 publications were retrieved. After reading the titles and abstracts, 51 reports of nonrandomized controlled studies and those that didn't use targeted therapy or repeated researches were excluded. By reading the full texts of the remaining publications, 13 randomized control studies [15–27] were included in accordance with the aforementioned inclusion and exclusion criteria (Figure 1). Of the 13 studies, 10 were used to compare the use of targeted drugs plus chemotherapy with pure chemotherapy. Three reports were used to investigate the efficiency of anti-EGFR *vs*. anti-VEGFR therapy in the conversion therapy for potentially CRLM. Five reports were referenced for the comparison of anti-EGFR MoAbs in mutant or wild RAS status in Figure 1.

The 13 studies comprised 7,520 cases in total, with 3,756 cases were from treatment groups and 3,764 from control groups. The characteristics of the included data are displayed in Table 1.

**Table 1:** RCT studies selecting conversion resection after conversion therapy

Study	Num	Regimens	ORR	R_0_ R	Jadad
RAISE[15]	536	FOLFIRI+Ramu	13.4	----	6
	536	FOLFIRI	12.5		
Ranhua 2015 [16]	65	FOLFIRI+Beva	47.7	----	7
	77	FOLFIRI	28.6		7
Marc 2010 [17]	303	FOLFIRI+Pani	40.0	----	5
	294	FOLFIRI	9.9		
Eric 2012 [18]	611	FOLFIRI+Afli	19.8	----	5
	605	FOLFIRI	11.1		
Le-Chi 2013 [19]	70	mFOLFOX6+Cetu	57.1	25.7	4
	68	mFOLFOX6	29.4	7.4	
Eric 2009 [20]	599	FOLFIRI+Cetu	46.9	5.1	7
	599	FOLFIRI	38.7	2.0	
Yuguo 2015 [21]	65	FOLFIRI+Pani+beva	46.2	----	5
	77	FOLFIRI	26.0		
Carsten 2009 [22]	113	FOLFOX4+Cetu	32.7	16.0	6
	120	FOLFOX4	22.5	4.3	
MAC CION [23]	362	CAP/FOLFOX/+Cetu	40.9	15.0	5
	367	CAP/FOLFOX	32.4	13.0	
PRIME [24]	326	FOLFOX4+Pani	54.9	8.3	6
	331	FOLFOX4	48.0	7.0	
FIRE-3 [25]	297	FOLFIRI+Cetu	62.0	----	7
	295	FOLFIRI+Beva	58.0		
PEAK [26]	139	mFOLFOX6+Pani	59.0	----	7
	139	mFOLFOX6+Beva	54.7		
CALGB/SWOG 80405 [27]	270	FOLFIRI/OX+Cetu	68.9		5
	256	FOLFIRI/OX+Beva	55.9		

### Main efficacy

#### Effect of targeted drugs *vs*. chemotherapy on ORR

**Figure 2:**
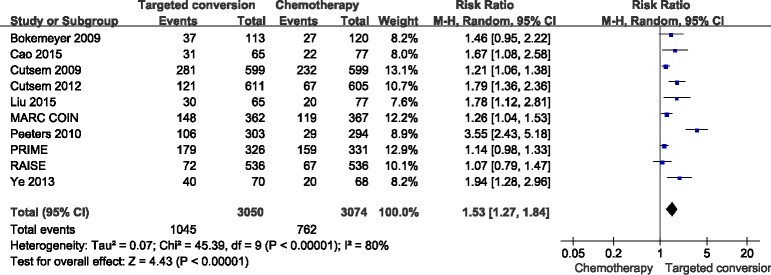
Comparison of targeted drugs plus chemotherapy with chemotherapy for CRLM patients in terms of the objective response rate (ORR)

ORR data for patients were provided in 10 studies [15–24]. The combined analysis of the 10 studies suggested great heterogeneity (*I*^2^ = 80%), and a combined RR for ORR of 1.53(95%CI: 1.27-1.84, *p* < 0.05) was obtained using a random-effects model (Figure 2).

### Effect of anti-EGFR *vs*. anti-VEGFR targeted chemotherapy on ORR

**Figure 3:**
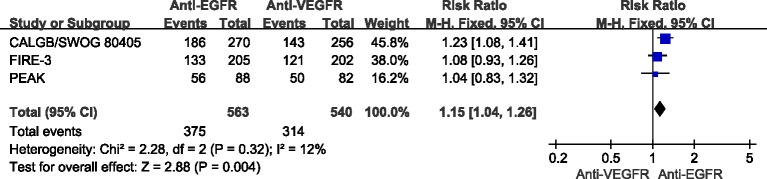
Comparison of anti-EGFR with anti-VEGFR targeted drug for CRLM patients with regard to the objective response rate (ORR)

ORR data for different MoAbs were provided in 3 studies [25–27]. The combined analysis of the 3 studies suggested mild heterogeneity (*I*^2^ = 12%), and a combined RR for ORR of 1.15 (95%CI: 1.04-1.26, *p* < 0.05) was obtained using a fixed-effects model (Figure 3).

### Anti-EGFR targeted drugs *vs*. chemotherapy

#### Different RAS/K-Ras status on ORR

**Figure 4:**
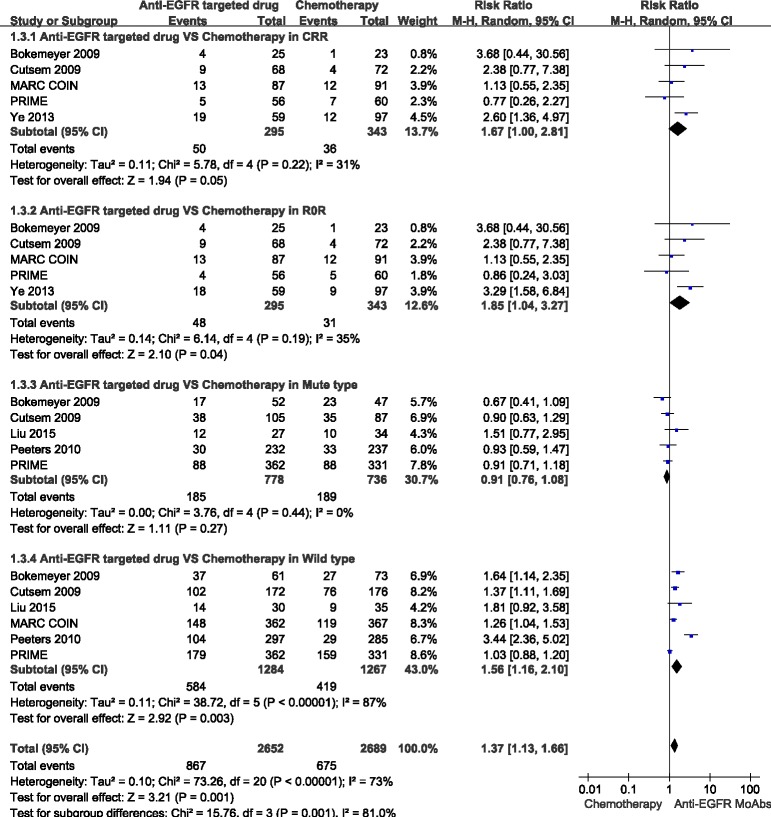
Comparison of anti-EGFR targeted drugs with simple chemotherapy: objective response rate (ORR), conversion resection rate (CRR), R resection rate (RR) for the CRLM patients in different RAS status

ORR data for anti-EGFR targeted chemotherapy in different patients with RAS/K-RAS status were provided in 5 studies [17, 20–22, 24]. In the mutant type RAS/K-RAS patients, the combined analysis of the 4 studies suggested mild heterogeneity (*I*^2^ = 20%), and a combined RR for ORR of 0.91 (95%CI: 0.77-1.08, *p* = 0.28) was obtained using a fixed-effects model. In the wild type RAS/K-RAS patients, the combined analysis of the 5 studies [17, 20–22, 24] suggested high heterogeneity (*I*^2^ = 87%), and a combined RR for ORR of 1.56(95%CI: 1.16-2.10, *p* < 0.05) was obtained using the random-effects model (Figure 4).

#### Conversion resection rates (CRR)

CRR data for anti-EGFR targeted chemotherapy were provided in five studies, and the combined analysis of these studies [19, 20, 22–24] suggested moderate heterogeneity (*I*^2^ = 31%), and a combined RR for CRR of 1.67 (95%CI: 1.00-2.81, *P* ≤ 0.05) was obtained using a random-effects model (Figure 4).

#### R0 resection rates (R0R)

R_0_R data for anti-EGFR targeted chemotherapy were provided in these studies, and the combined analysis of the 5 studies [19, 20, 22–24] indicated moderate heterogeneity (*I*^2^ = 35%), and a combined RR for R_0_R of 1.85(95%CI: 1.04-3.27, *p* < 0.05) was obtained using a random-effects model (Figure 4).

#### Sensitivity analysis

Every study was excluded each time to assess the effect of the individual data set to the pooled RRs, and studies with high heterogeneity were subsequently removed.

#### Publication bias

Publication bias was determined by Begg's funnel plot and the Egger linear regression test to detect the funnel plot asymmetry. If the Egger test calculated *p* < 0.05, publication bias was assessed to exist. Our results demonstrated that there was no evident publication bias in the present meta-analysis.

## DISCUSSION

The primary aim of treatment for the CRLM is conversion to resectable as it provides the only possibility for cure [28]. A staging system was proposed by the European Colorectal Metastases Treatment Group system that divides the CRLM into 4 groups. These include M_0_: no metastases; M_1a_: resectable liver metastases; M_1b_: potentially resectable liver metastases; and M_1c_: liver metastases that are unlikely to ever be resectable. For M_1a_ resectable patients and M_1b_ patients who qualify as resectable after systemic treatment, resection offers the possibility of a cure. For the M_1c_ group, the possibility of resection should not be excluded and each case should be considered individually [8]. In conversion therapy, common combined chemotherapy regimens include FOLFIRI [21], FOLFOX [22] and CAPOX [23]. However, the efficiency of such chemotherapy is only 30-40%, even in first line application. Also the addition of anti-VEGFR/EGFR agents could improve both OS and the rates of secondary resection [21–23]. The present study confirmed the conversion efficiency of different MoAbs plus chemotherapy in patients with wild or mutant type RAS. The study was also able to ascertain from data concerning the ORR, that conversion therapy with MoAbs(anti-EGFR or anti-VEGFR) was more efficacious than simple chemotherapy. When comparing the two types of MoAbs therapy, anti-EGFR elicited a better response. From the study, patients with wild type RAS could benefit from anti-EGFR conversion therapy. However, there was no observed benefit for patients with the mutant type RAS considering whether MoAbs were administered. The underlying mechanisms involve RAS proteins, which are GTPases and molecular switches for a variety of critical cellular activities involving K-RAS, N-RAS, and H-RAS in the EGFR signaling pathway. Their function is tightly monitored in normal cells but the oncogenic mutation of the RAS gene, which creates constitutively active RAS proteins, result in uncontrolled proliferation or survival in tumor cells and resistance to anti-EGFR drugs[29]. Therefore, the wild type RAS patients were included to evaluate the overall conversion resection and R_0_R following treatment with anti-EGFR MoAbs plus chemotherapy. Both R_0_ resection and overall resection rate indicated the advantages of anti-EGFR MoAbs in conversion therapy. Certain factors were not considered in the study design of the present meta-analysis. For patients with RAS and BRAF mutations, triplet chemotherapy and combinations with bevacizumab represented two rational alternatives on the basis of high response rates and tumor shrinkage [30]; however their efficacy in the conversion setting has yet to be confirmed in specifically designed studies. The gene type of RAS analysis in this study were included both RAS and K-RAS for the technology of detecting RAS was created in the recent years and a part of RCTs only described the K-RAS type. In addition, not all the studies selected presented the resection rate or R_0_ resection but only the ORR and unequivocal on ORR and resection rate in potentially resectable patients are still required in order for the heterogeneity to be amplified.

In clinical trials regarding adverse effects, it has been revealed that chemotherapy may induce liver damage; in particular oxaliplatin has been associated with sinusoidal wall disruption, resulting in sinusoidal obstruction (SOS) [32]. Similarly, irinotecan can induce a form of non-alcoholic fatty liver disease known as steatohepatitis [33]. Therefore, in order to decrease these and improve the therapeutic efficiency for patients with colorectal cancer, molecularly targeted therapies have been developed [34], among which, the rates reported are 43-81% with the addition of the molecular targeted drug bevacizumab or cetuximab and panitumumab [35]. Molecular targeted drugs expand specific changes in tumor cells to achieve relative target specificity. This not only enhances the anti-cancer effect, but also reduces the damage to normal cells.

In conclusion, the present study demonstrates a clear incentive to select MoAbs for the treatment of potentially resectable CRLM patients with different RAS/K-RAS status. MoAbs proffer additional benefits in conversion therapy compared to the simple chemotherapy. Furthermore, patients excluded mutant type RAS/K-RAS could benefit from MoAbs for the conversion therapy and among the different MoAbs, anti-EGFR therapies have greater efficacy compared to anti-VEGFR, however, this is only the case for patients with wild type RAS/K-RAS.

## SUPPLEMENTARY MATERIALS



## References

[1] Torre L.A, Bray F, Siegel R.L, Ferlay J, Lortet-Tieulent J, Jemal A (2015). Global Cancer Statistics, 2012. CA Cancer J Clin.

[2] Van Cutsem E, Nordlinger B, Cervantes A, E.G.W. Group (2010). Advanced colorectal cancer: ESMO Clinical Practice Guidelines for treatment. Ann Oncol.

[3] Veereman G, Robays J, Verleye L, Leroy R, Rolfo C, Van Cutsem E, Bielen D, Ceelen W, Danse E, De Man M, Demetter P, Flamen P, Hendlisz A (2015). Pooled analysis of the surgical treatment for colorectal cancer liver metastases. Crit Rev Oncol Hematol.

[4] Kanas G.P, Taylor A, Primrose J.N, Langeberg W.J, Kelsh M.A, Mowat F.S, Alexander D.D, Choti M.A, Poston G (2012). Survival after liver resection in metastatic colorectal cancer: review and meta-analysis of prognostic factors. Clin Epidemiol.

[5] Grothey A, Van Cutsem E, Sobrero A, Siena S, Falcone A, Ychou M, Humblet Y, Bouche O, Mineur L, Barone C, Adenis A, Tabernero J, Yoshino T (2013). Regorafenib monotherapy for previously treated metastatic colorectal cancer (CORRECT): an international, multicentre, randomised, placebo-controlled, phase 3 trial. Lancet.

[6] Kemeny N (2007). Presurgical chemotherapy in patients being considered for liver resection. Oncologist.

[7] Marino D, Leone F, D'Avanzo F, Ribero D, Capussotti L, Aglietta M (2014). Potentially resectable metastatic colorectal cancer: an individualized approach to conversion therapy. Crit Rev Oncol Hematol.

[8] Nordlinger B, Van Cutsem E, Rougier P, Kohne C.H, Ychou M, Sobrero A, Adam R, Arvidsson D, Carrato A, Georgoulias V, Giuliante F, Glimelius B, Golling M (2007). Does chemotherapy prior to liver resection increase the potential for cure in patients with metastatic colorectal cancer? A report from the European Colorectal Metastases Treatment Group. European Journal of Cancer.

[9] Li D.B, Ye F, Wu X.R, Wu L.P, Chen J.X, Li B, Zhou Y.M (2013). Preoperative administration of bevacizumab is safe for patients with colorectal liver metastases. World Journal of Gastroenterology.

[10] Khan K, Wale A, Brown G, Chau I (2014). Colorectal cancer with liver metastases: neoadjuvant chemotherapy, surgical resection first or palliation alone?. World J Gastroenterol.

[11] Kishi Y, Zorzi D, Contreras C.M, Maru D.M, Kopetz S, Ribero D, Motta M, Ravarino N, Risio M, Curley S.A, Abdalla E.K, Capussotti L, Vauthey J.N (2010). Extended preoperative chemotherapy does not improve pathologic response and increases postoperative liver insufficiency after hepatic resection for colorectal liver metastases. Ann Surg Oncol.

[12] Worni M, Shah K.N, Clary B.M (2014). Colorectal cancer with potentially resectable hepatic metastases: optimizing treatment. Curr Oncol Rep.

[13] Gerlinger M, Rowan A.J, Horswell S, Larkin J, Endesfelder D, Gronroos E, Martinez P, Matthews N, Stewart A, Tarpey P, Varela I, Phillimore B, Begum S (2012). Intratumor heterogeneity and branched evolution revealed by multiregion sequencing. N Engl J Med.

[14] Jadad A.R, Moore R.A, Carroll D, Jenkinson C, Reynolds D.J, Gavaghan D.J, McQuay H.J (1996). Assessing the quality of reports of randomized clinical trials: is blinding necessary?. Control Clin Trials.

[15] Tabernero J, Takayuki Y, Cohn A.L (2015). Ramucirumab *versus* placebo in combination with second-line FOLFIRI in patients with metastatic colorectal carcinoma that progressed during or after first-line therapy with bevacizumab, oxaliplatin, and a fluoropyrimidine (RAISE): a randomised, double-blind, multicentre, phase 3 study (vol 16, pg 499, 2015). Lancet Oncology.

[16] Cao R.H, Zhang S, Ma D.D, Hu L.K (2015). A multi-center randomized phase II clinical study of bevacizumab plus irinotecan, 5-fluorouracil, and leucovorin (FOLFIRI) compared with FOLFIRI alone as second-line treatment for Chinese patients with metastatic colorectal cancer. Medical Oncology.

[17] Peeters M, Price T.J, Cervantes A, Sobrero A.F, Ducreux M, Hotko Y, Andre T, Chan E, Lordick F, Punt C.J.A, Strickland A.H, Wilson G, Ciuleanu T.E (2010). Randomized Phase III Study of Panitumumab With Fluorouracil, Leucovorin, and Irinotecan (FOLFIRI) Compared With FOLFIRI Alone As Second-Line Treatment in Patients With Metastatic Colorectal Cancer. Journal of Clinical Oncology.

[18] Van Cutsem E, Tabernero J, Lakomy R, Prenen H, Prausova J, Macarulla T, Ruff P, van Hazel G.A, Moiseyenko V, Ferry D, McKendrick J, Polikoff J, Tellier A (2012). Addition of Aflibercept to Fluorouracil, Leucovorin, and Irinotecan Improves Survival in a Phase III Randomized Trial in Patients With Metastatic Colorectal Cancer Previously Treated With an Oxaliplatin-Based Regimen. Journal of Clinical Oncology.

[19] Ye L.C, Liu T.S, Ren L, Wei Y, Zhu D.X, Zai S.Y, Ye Q.H, Yu Y.Y, Xu B, Qin X.Y, Xu J.M (2013). Randomized Controlled Trial of Cetuximab Plus Chemotherapy for Patients With KRAS Wild-Type Unresectable Colorectal Liver-Limited Metastases. Journal of Clinical Oncology.

[20] Van Cutsem E, Kohne C.H, Hitre E, Zaluski J, Chien C.R.C, Makhson A, D'Haens G, Pinter T, Lim R, Bodoky G, Roh J.K, Folprecht G, Ruff P (2009). Cetuximab and Chemotherapy as Initial Treatment for Metastatic Colorectal Cancer. New England Journal of Medicine.

[21] Liu Y.G, Luan L.J, Wang X.L (2015). A randomized Phase II clinical study of combining panitumumab and bevacizumab, plus irinotecan, 5-fluorouracil, and leucovorin (FOLFIRI) compared with FOLFIRI alone as second-line treatment for patients with metastatic colorectal cancer and KRAS mutation. Oncotargets and Therapy.

[22] Bokemeyer C, Bondarenko I, Makhson A, Hartmann J.T, Aparicio J, de Braud F, Donea S, Ludwig H, Schuch G, Stroh C, Loos A.H, Zubel A, Koralewski P (2009). Fluorouracil, Leucovorin, and Oxaliplatin With and Without Cetuximab in the First-Line Treatment of Metastatic Colorectal Cancer. Journal of Clinical Oncology.

[23] Maughan T.S, Adams R.A, Smith C.G, Meade A.M, Seymour M.T, Wilson R.H, Idziaszczyk S, Harris R, Fisher D, Kenny S.L, Kay E, Mitchell J.K, Madi A (2011). Addition of cetuximab to oxaliplatin-based first-line combination chemotherapy for treatment of advanced colorectal cancer: results of the randomised phase 3 MRC COIN trial. Lancet.

[24] Douillard J.Y, Siena S, Cassidy J, Tabernero J, Burkes R, Barugel M, Humblet Y, Bodoky G, Cunningham D, Jassem J, Rivera F, Kocakova I, Ruff P (2010). Randomized, Phase III Trial of Panitumumab With Infusional Fluorouracil, Leucovorin, and Oxaliplatin (FOLFOX4) *Versus* FOLFOX4 Alone As First-Line Treatment in Patients With Previously Untreated Metastatic Colorectal Cancer: The PRIME Study. Journal of Clinical Oncology.

[25] Heinemann V, von Weikersthal L.F, Decker T, Kiani A, Vehling-Kaiser U, Al-Batran S.E, Heintges T, Lerchenmuller C, Kahl C, Seipelt G, Kullmann F, Stauch M, Scheithauer W (2014). FOLFIRI plus cetuximab *versus* FOLFIRI plus bevacizumab as first-line treatment for patients with metastatic colorectal cancer (FIRE-3): a randomised, open-label, phase 3 trial. Lancet Oncology.

[26] Schwartzberg L.S, Rivera F, Karthaus M, Fasola G, Canon J.L, Hecht J.R, Yu H, Oliner K.S, Go W.Y (2014). PEAK: A Randomized, Multicenter Phase II Study of Panitumumab Plus Modified Fluorouracil, Leucovorin, and Oxaliplatin (mFOLFOX6) or Bevacizumab Plus mFOLFOX6 in Patients With Previously Untreated, Unresectable, Wild-Type KRAS Exon 2 Metastatic Colorectal Cancer. Journal of Clinical Oncology.

[27] Venook A.P, Niedzwiecki D, Lenz H.J, Innocenti F, Mahoney M.R, O'Neil B.H, Shaw J.E, Polite B.N, Hochster H.S, Atkins J.N, Goldberg R.M, Mayer R.J, Schilsky R.L (2014). CALGB/SWOG 80405: Phase III trial of irinotecan/5-FU/leucovorin (FOLFIRI) or oxaliplatin/5-FU/leucovorin (mFOLFOX6) with bevacizumab (BV) or cetuximab (CET) for patients (pts) with KRAS wild-type (wt) untreated metastatic adenocarcinoma of the colon or rectum (MCRC). Journal of Clinical Oncology.

[28] Jones R.P, Hamann S, Malik H.Z, Fenwick S.W, Poston G.J, Folprecht G (2014). Defined criteria for resectability improves rates of secondary resection after systemic therapy for liver limited metastatic colorectal cancer. European Journal of Cancer.

[29] Huang L, Fu L (2015). Mechanisms of resistance to EGFR tyrosine kinase inhibitors. Acta Pharm Sin B.

[30] Salvatore L, Loupakis F, Cremolini C, Schirripa M, Masi G, Antoniotti C, Fornaro L, Sensi E, Lupi C, Bergamo F, Lonardi S, Zagonel V, Fontanini G (2012). FOLFOXIRI plus bevacizumab as first-line treatment of BRAF-mutant metastatic colorectal cancer patients. Journal of Clinical Oncology.

[31] Akgul O, Cetinkaya E, Ersoz S, Tez M (2014). Role of surgery in colorectal cancer liver metastases. World Journal of Gastroenterology.

[32] Aloia T, Sebagh M, Plasse M, Karam V, Levi F, Giacchetti S, Azoulay D, Bismuth H, Castaing D, Adam R (2006). Liver histology and surgical outcomes after preoperative chemotherapy with fluorouracil plus oxaliplatin in colorectal cancer liver metastases. Journal of Clinical Oncology.

[33] Fernandez F.G, Ritter J, Goodwin J.W, Linehan D.C, Hawkins W.G, Strasberg S.M (2005). Effect of steatohepatitis associated with irinotecan or oxaliplatin pretreatment on resectability of hepatic colorectal metastases. Journal of the American College of Surgeons.

[34] Venook A (2005). Critical evaluation of current treatments in metastatic colorectal cancer. Oncologist.

[35] Sugihara K, Uetake H (2012). Therapeutic strategies for hepatic metastasis of colorectal cancer: overview. Journal of Hepato-Biliary-Pancreatic Sciences.

